# New products

**DOI:** 10.1155/S1463924689000118

**Published:** 1989


Journal of Automatic Chemistry Vol. 11, No. 1 (January–February 1989), pp. 44–54

### New products

### Hewlett-Packard particle-beam LC/MS system

Hewlett-Packard have introduced a particle beam (PB) LC/MS system that routinely generates library-matchable electron-impact spectra of non-volatile and thermally labile compounds.

The EI spectra can be checked against standard or custom spectral libraries for positive compound identification. Chemical ionization spectra can also be produced.

HP say that this PB LC/MS system is easier to use than any previous LC/MS system. PB interface operation requires one simple adjustment, and little modification is required of either HPLC or MS methodology.

The HP PB LC/MS interface is derived from the original MAGIC particle-beam interface invented at Georgia Institute of Technology, which has granted HP exclusive rights to further develop and market this invention.

A complete HP PB LC/MS system consists of the particle beam interface mounted on the HP 5988A MS, an LC (either the integrated HP 1090 or the modular HP 1050) and a data system (either the HP 59970C ChemStation for single instrument operation or the HP 1000 RTE A-series for multi-instrument, multitasking or multiuser operation). In addition, the HP PB LC/MS interface is available separately to be field-installed as a retrofit on HP 5988A and 5987A MS instruments.

The HP PB LC/MS uses the same switchable EI/CI ion source and the same software and data systems that are used for HP 5988A GC/MS. When a GC is added, creating a versatile PB LC/GC/MS, the system can be instantly switched from LC/MS to GC/MS.

In addition to GC/MS, other HP 5988A options available for the HP PB LC/MS system included: thermospray, DCI, FAB, direct insertion probe, packed- or capillary-column operation, and 10 to 1,000 or 10 to 2,000 amu operation.

The HP PB LC/MS greatly increases the chemist's ability to analyse environmental compounds, pharmaceuticals, drugs of abuse, foods and life science processes. Potential applications include most compounds that can be analysed by either GC or LC.

*For further information, contact: Hewlett-Packard SA, Analytical Products Group, Route du Nant-d'Avril 150, P.O. Box, CH-1217 Meyrin 2, Geneva, Switzerland.*

### TLC and GC/MS determination of 40 common drugs of abuse

Hewlett-Packard Company and Marion Laboratories Inc. have published a 100-page monograph *Screening and Confirmation of Drugs with Toxi-Lab TLC and Hewlett-Packard GC/MS Systems.*

The free booklet presents step by step methods and procedures for determination of 40 common drugs of abuse including antidepressants, benzodiazepine, pentazocine and tripelennamine. The booklet, containing many full-colour illustrations and a list of references, explains the interpretation of results.

*For further information, contact: Hewlett-Packard SA, Route du Nant-d'Avril 150, P.O. Box CH-1217 Meyrin 2, Geneva, Switzerland.*

### Mass spectrometer uses new technology

The Spectramass PC 2000 is a high performance quadrupole mass analyser aimed at the precision end of the residual gas analysis market as well as providing the flexibility necessary to meet the requirements of process control applications.

The heart of the mass spectrometer is a new ion source which has a built-in independent total pressure collector. Rapid switching between partial and total pressure measurement allows software normalisation of the spectrum by ensuring that the sum of the partial pressures is always equal to that of the total pressure measurement. This technique not only greatly improves accuracy but eliminates non-linearity errors at high pressures and loss of sensitivity caused by contamination.

Software advances are much enhanced by the use of high resolution colour graphics which, together with five button keypad or mouse operation, make the comprehensive software very easy to use.

*For further information, contact: Spectramass Ltd, Radnor Park Industrial Estate, Congleton, Cheshire CW12 4XR, UK. Tel.: 0260 279531.*

### Simulated distillation PC software package for Perkin-Elmer 8000 series gas chromatographs

Simulated Distillation software is the latest applications software package available for Perkin-Elmer 8000 Series GCs (Models 8400 and 8500). Designed primarily to produce boiling-point distribution data for gasolines and distillates according to ASTM D3710 and ASTM D2887, the package incorporates an easy to use menu-driven user interaction.

The integral data handling of the 8000 Series is used to carry out routine data handling calculations, while a PC running IBM PC DOS or MS DOS performs the Simulated Distillation calculations.

Separate functions allow boiling-point and response factor calibrations, sample analysis, system performance checks, the ability to download disk-stored methods for setting up the GC and data handling parameters and the facility to estab-

0142–0453/89 $3.00 © 1989 Taylor & Francis Ltd.

lish a database comprising physical constants pertinent to samples to be analysed.

The software is fully compatible with the AS-8300 liquid autosampler for full automation and unattended operation.

*For further information, contact: Perkin-Elmer Limited, Post Office Lane, Beaconsfield, Bucks HP9 1QA, UK. Tel.: 0494 676161.*

### Diode array detector for HPLC

The Model LC-480 Autoscan from Perkin-Elmer is a high performance diode array detector for HPLC. It combines high sensitivity and excellent flow cell characteristics with high spectral resolution at low analyte levels.

The LC-480 can operate as a stand-alone detector offering multichannel detection and arithmetic signal combinations to perform purity checks or spectral suppression techniques. Spectra are acquired automatically or manually and can be plotted in various formats on a printer-plotter.

Alternatively, installation of a computer interface allows transfer of chromatograms in the form of entire UV spectra to an IBM-compatible PC for data manipulation. Chromatograms may be selected at any wavelength and spectra at any time. Spectral manipulation for overlaying, differentiation, subtraction or library functions are available for development of chromatography or for investigative trouble shooting. Similarly, chromatograms may be compared with standards and integration facilities allow quantitative analysis.

Specific data reduction/evaluation regimes may be implemented automatically with macro language to simplify use.

The LC-480 + PC therefore aids research and development in, for example, the investigation of anomalies highlighted in the routine environment by such on-line 'smart' detectors as the LC-235/135.

*For further information, contact: Perkin-Elmer Limited, Post Office Lane, Beaconsfield, Bucks HP9 1QA, UK. Tel.: 04946 6161.*

### Brochure describes quantitative method development and multi-component analysis

Hewlett-Packard Company has published a free 10-page brochure (Publication 5956-4172) describing how to use HP equipment to take full advantage of diode-array UV/visible spectroscopy for the development of quantitative methods and for multi-component analysis.

For single-component analysis, the brochure describes various task modes used to provide answers to the following questions: Which wavelengths provide the best calibration curve? How accurate is the method for 'real' samples? Is the sample pure? How reproducible is the method?

For multicomponent analysis, the brochure states that concentrations for up to 12 components can be determined. Illustrated examples are presented to show how the fast spectral data acquisition and wavelength reproducibility of the diode-array spectrophotometer provide the basis for improved calculation algorithms and better results.

The HP 89511A quantitative software package described in the brochure is designed to be used with the HP 8452A diode-array UV/visible spectrophotometer and the HP UV/visible ChemStation.

*For further information, contact: Hewlett-Packard SA, Route du Nant-d'Avril 150, P.O. Box CH-1217 Meyrin 2, Geneva, Switzerland.*

### Perkin-Elmer Plasma 40 Wear Metals Analyzer

A new high performance inductively coupled plasma (ICP) atomic emission spectrometer introduced by Perkin-Elmer is optimized for the determination of wear metals in oil samples. The Plasma 40 Wear Metals Analyzer (P40 WMA) targets concentration trends of wear metals in an engine's lubricating oil which can be directly related to specific engine components.

The P40 WMA incorporates all the important features of the highly successful Plasma 40 ICP emission spectrometer, including a free-running RF generator, advanced software features, instrument control from a PC and an integrated bench-top design. In addition, the sample introduction system has been designed to withstand organic solvents such as xylene or kerosene. Both the software and the optical system have also been optimized for easy and rapid determination of wear metals in oil.

*For further information, or an applications paper detailing performance results, contact: Perkin-Elmer Limited, Post Office Lane, Beaconsfield, Bucks HP9 1QA, UK. Tel.: 04946 6161.*

### Guide to techniques and applications of atomic spectroscopy

Perkin-Elmer Limited has produced a *Guide to Techniques and Applications of Atomic Spectroscopy* which provides a quick reference to the major atomic spectroscopy techniques and their application to analytical problems.

Included is a section on the fundamentals of atomic spectroscopy, a bibliography listing selected articles on various applications, and a description of Perkin-Elmer's complete line of atomic spectroscopy instrumentation. Reprints of the articles listed in the bibliography are available free of charge.

*For further information, contact: Perkin-Elmer Limited, Post Office Lane, Beaconsfield, Bucks HP9 1QA, UK. Tel.: 0494 676161.*

### Radiometer expands TitraLab series

Radiometer Analytical A/S has expanded the TitraLab series with the launch of the TIM90 Titration Manager. The TIM90 Titration Manager will directly input balance information, drive a multiple burette station and operate in four titration modes. It can perform not only simple but also complex titrations involving several reagents or electrodes, e.g. acid-base, redox and complexometric titrations with up to four equivalence points.

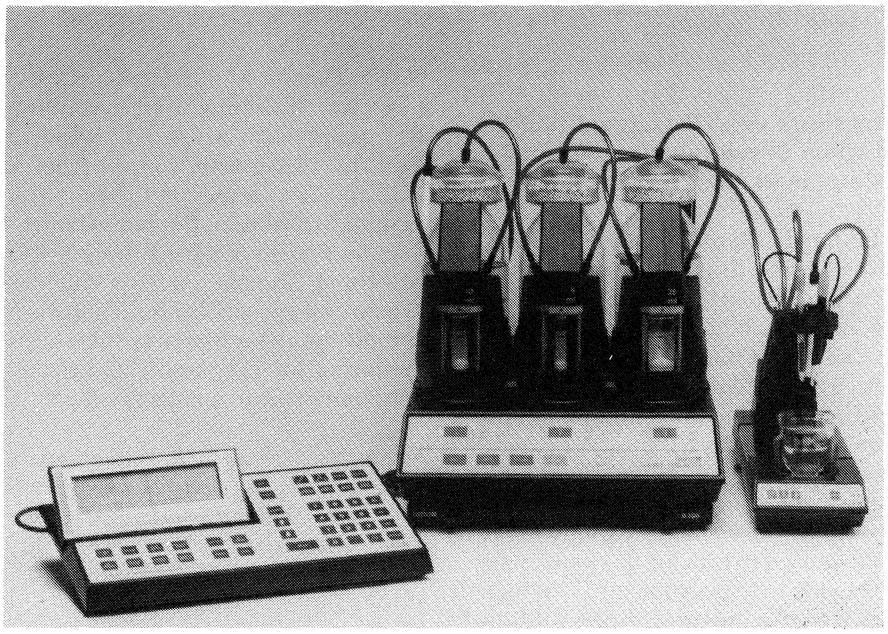

*TitraLab 3 titration laboratory from Radiometer incorporating the new TIM90 Titration Manager, an ABU93 Triburette and a SAM90 Sample Station.*

Operator communication is via a dedicated keyboard which has specific single-function keys and a large graphic LCD display with eight information lines. This, together with the live titration curve, provides the user with a continuous overview of what is happening. After titration, curves can be reviewed on the screen and, if appropriate, new parameters incorporated to secure correct results. Post-titration derivative curves are also available on the display, and results, titration points, curves and method data can all be printed for permanent records. A secure code-protected and battery-buffered memory provides storage and access for 40 titration methods, 16 sets of titrant data and up to 64 groups of sample information.

Radiometer sees the TIM90's chief application as the brain of a TitraLab titration laboratory performing various routine analyses in, for instance, quality control laboratories. It is easily positioned in front, or to the side, of an autoburette and sample station. The ABU93 Triburette has three built-in burette drives and two electrode input channels. The SAC80 Sample Changer allows unattended analysis of up to 20 samples in one cycle, every beaker utilising its own titration procedure.

The TIM90 Titration Manager is supplied as standard with all interfaces for printer/plotter, balance, sample changer and external keyboard, thus providing facilities from mass input to results printout avoiding transcription errors, speeding-up of batch analysis and even connection of a robotic system.

*For further information, contact: Analytical Division, Radiometer Ltd, The Manor, Manor Royal, Crawley, West Sussex RH10 2PY, UK. Tel.: 0293 517599.*

### New balance speeds finite weighing operation

Sartorius Limited has introduced a new top-loading micro-balance. The M3P electronic top-loading micro-balance offers a total capacity of 3 g including the tare range which is 1.5 g. The micro-balance has three automatically selectable ranges of 0–500, 0–1000 and 0–1500 mg and can be read to an accuracy of 1 μg when required, although other ranges include 2 and 5 μg.

As well as the simplified pan loading, the M3P eliminates the need to arrest and release the weighing system as is necessary with other micro-balances in this weighing range.

A number of features are built-in as standard. These include an effective electronic vibration filtering system, semi-automatic calibration and extensive thermal shielding of the housing, features combining to give the user confidence in obtaining accurate results.

All controls are contained within a compact control panel mounted on the front of the balance. Use of latest electronic technology has given a control system which guides the user through every step of a weighing sequence such as taring, use of internal compensating weights and calibration. The micro-balance housing measures 219 × 153 × 219 mm and the top-loading pan has a diameter of 22 mm.

*For further information, contact: Sartorius Limited, 18 Avenue Road, Belmont, Surrey SM2 6JD, UK. Tel.: 01 642 8691.*

### Liquid dispensing system features 'self-teach' programming

A bench-top liquid dispensing system that is easy to program and provides high speed, continuous path coverage has been introduced by Automation Unlimited of Woburn, Massachusetts, USA.

The Automation Unlimited LD 1212 Programmable Motion Controlled Positioner is a fully automatic three-axis liquid dispensing system that features 'self-teach' digitized programming. Utilizing *X*, *Y* and *Z* joysticks and 10 user-assignable spot, line and pattern function keys, programmed commands include point-to-point, linear or circular motion, feed rates, dwell times, mirror image, hold, step and repeat, automatic home and floating zero.

Equipped with an RS232C interface which allows part program transfer, the liquid dispensing system operates at speeds up to 500 in/min (200 mm/s) with ± 0.002 in (0.051 mm) repeatability and provides 12 × 12 in (305 mm × 305 mm) *X-Y* travel with 2 in (5.08 cm) *Z* travel. A syringe dispenser is standard and optional pinch or shot valves are available.

*For further information, contact: Automation Unlimited, 134 New Boston St.,*

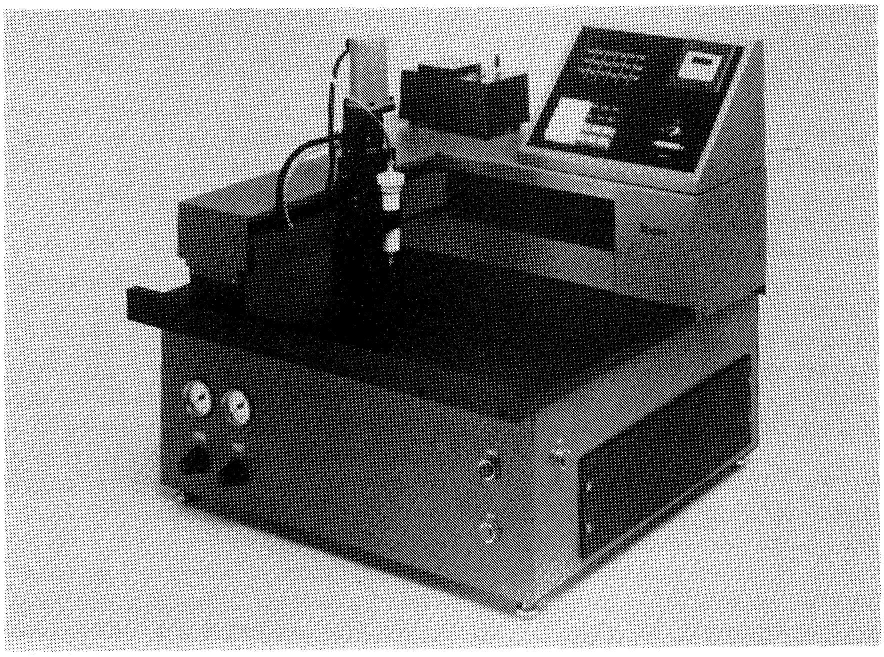

*Automation Unlimited LD 121 Programmable Motion Controlled Positioner liquid dispensing system.*

*Woburn, MA 01801, USA. Tel.: 617 933 7288.*

### Disposable filtration capsules

Whatman are now marketing Polycap HD, a disposable filtration capsule which consists of a polypropylene housing and polypropylene non-woven depth filter. The capsule offers a high loading capacity, high filtration efficiency and low differential pressure, whilst also having a wide pH, pressure and temperature range and a long life. The use of a special polypropylene depth filter ensures that the loading capacity of the filter is increased without compromising the particle retention, and the differential pressure is kept to a minimum.

These features make the Polycap HD suitable for the filtration of larger volumes of liquids or gases, the filtration of liquids or gases with a high particle density and large volumes of solutions where non-specific binding must be avoided. This is a problem when filtering some biological solutions using either glass or membrane filtration. The larger particle retention makes the Polycap HD ideal for any pre-filtration applications, thereby increasing the life time of any down-stream filtration media.

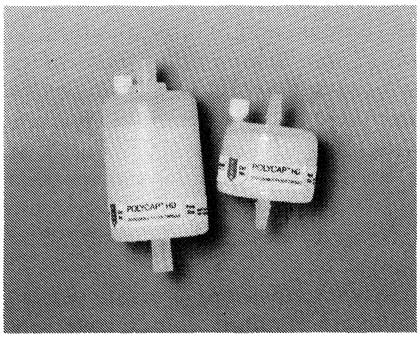

*Whatman Polycap HD 36 and 75 disposable filtration capsules.*

During the manufacture of the capsule no mould-releasing agents, powdered materials or adhesives are used. This reduces contamination by material that may leach into the filtrate, and enhances the structural strength.

Because the filter media is also polypropylene, the unit has a very wide chemical compatibility range. This is an advantage over some membrane filtration media where the chemical compatibility may be limited, and glass microfibre filtration where dissolution of the glass or ionic impurities may be a problem.

The unit can be autoclaved as many as 50 times without any significant loss in particle retention or filtration efficiency. A complete list of chemical compatibilities and instructions are given in a leaflet that accompanies the product.

Also available is the Polycap TF, a disposable encapsulated membrane filtration capsule that incorporates a fully hydrophobic membrane in a polypropylene housing. This combination of materials, with its large filtration area, make this capsule ideal for larger scale filtration of aggressive chemical solvents. Typically, up to 20 l/min of water may be filtered by the larger capsules at a pressure of only 1 bar, making this capsule ideal for the rapid filtration of large amounts of solution, e.g. preparative HPLC, larger scale bioreactors.

The Polycap TF may be autoclaved and will withstand temperatures in excess of 130 °C. This enables the 0.1 and 0.2 $\mu$m pore size capsules to be used for filtration sterilization, the smaller pore size being used for 'ultraclean' applications. The 0.45 $\mu$m pore size capsule is routinely used for filtration of large scale HPLC solvents. The largest pore size capsule, 1.0 $\mu$m, incorporates a polypropylene monofilament pre-filter. As a result, this capsule has a much higher loading capacity, yet retains the advantages of membrane filtration. Accordingly it is ideal for the large scale filtration of solutions that are likely to block ordinary membrane filters, e.g. protein solutions.

*For further information, contact: Whatman Ltd, Springfield Mill, Maidstone, Kent ME14 2LE, UK. Tel.: 0622 692022.*

### Micro-cuvette capability for WPA colorimeters

All three models in the CO200 series can now handle micro-cuvettes, allowing smaller samples to be used with consequent savings in reagents.

The new micro-cuvette capability allows the CO200 Series to operate on sample volumes as small as 0.5 $cm^3$ using $10 \times 3$ mm micro-cuvettes; conventional 10 mm square section cuvettes can still be used in the instruments if required. The dual cuvette chamber is fitted with a stray light cover and holds both the reference and the sample.

*For further information, contact: WPA, The Old Station, Linton, Cambridge CB1 6NW, UK. Tel.: 0223 892688*

### MTS puts industrial colour analysis on an IBM PC

By coupling a spectrophotometer to an IBM compatible PC and a high resolution colour monitor, MTS Colorimétrie has created a powerful tool for analysing and comparing colours. The system's software makes it specifically useful for solving manufacturing problems in the ink and paint industries and in other fields such as plastics and textiles.

The MTS unit is based on the company's own spectrophotometer, but the key feature of this system is the software, which is available in French, English and German versions and can also be used to drive other spectrophotometers. It is presented as a set of independent modules each of which can be called from a general menu. There is a basic colorimetry module with an integrated sorting facility. This allows you to measure, compare and sort colour samples in a range from 400 to 710 nm.

There is a module for characterising the pigment or colour base: it calculates $K$-absorption and $s$-diffusion every 20 nm according to the degree of concentration. It is also possible to analyse the colouring strength which is calculated on 32 points of the spectrum.

Other features include the ability to derive formulae for colour shades by combining pigments in different concentrations either manually or automatically. Up to 65000 colour formulae can then be stored and retrieved using the system's integrated database. These features constitute a unique aid to colour analysis both for industrial production and in the laboratory.

*For further information, contact: MTS Colorimétrie, Immeuble Jules César 12, chaussée Jules César, F-95520 Osny, France. Tel.: 331 30 735220.*

### New literature on quality hydraulically-powered pumps for clean oil filling systems

Lindley Flowtech Ltd has just announced new literature about its range of hydraulically-powered clean oil filling pumps for use in mines and other hostile environments.

The range of reliable, British Coal approved pumps is capable of handling oils ranging from hydraulic to heavy gear oil. The four types available cater for virtually every need.

The Fast Flow Pump offers the most flexible solution to filling reservoirs and tanks at speed. Producing enough power to operate through a blocked in-line filter, the dual-powered unit comfortably delivers up to 15 gpm when driven hydraulically. Even when manually operated, in the absence of hydraulic power, it delivers at up to 10 gpm.

The Medium-Duty Pump is a versatile, compact and reliable unit built to a high specification. Flow rates of up to 6 gpm make it ideally suited to machine-mounted clean fill systems. Operating at up to 400 lb in$^{-2}$, it transfers both hydraulic and gear oils with ease through a rotary selector valve or a flexible hose system.

Wherever enhanced pressure performance is required, the Heavy-Duty Pump can be relied on to supply oil to machines at distances of up to 300 m. Normal operating pressure is up to 1000 lb in$^{-2}$ with a maximum flow rate up to 3 gpm.

The Light-Duty Pump is a small unit which can be machine-mounted in even the most confined spaces. Rated at 5 gpm (max), at up to 400 lb in$^{-2}$, it is ideal for the more compact machine-centered transfer systems.

Unless otherwise specified, all Lindley Flowtech pumps are equipped with a unique, tamper-proof device to prevent pump damage through flow and pressure excesses, thus providing complete operational safety at motor input pressures of up to 5500 lb in$^{-2}$.

Lindley Flowtech's full technical consultancy is backed by an on-site fitting and maintenance service while quality assurance to Lloyd's/ISO 9001 leaves nothing to chance.

*For further information, please contact: Lindley Flowtech Ltd, Flowtech House, Campus Road, Bradford, West Yorkshire BD7 1HR, UK. Tel.: 0274 723454.*

### Bayer substrate helps clean up waste water

As part of its environmental protection research programme, Bayer has developed a polyurethane based carrier for use as a substrate for the growth of micro-organisms in waste water treatment plants.

Applications envisaged include anaerobic treatment of low degradability effluents produced by the pulp, paper, leather and textile industries, and polluted waste water from the food and beverage industries.

Bayer carried out a series of successful tests on the use of the PU substrate with potential industrial users and has now started semi-commercial production.

As a result of these pilot plant trials, Bayer foresees many advantages for biological waste water treatment. Together with shorter treatment times, these benefits allow a considerable reduction in reactor volumes, leading to savings in capital investment costs for new plant, and increased capacities from retrofitting existing plants.

*For further information, contact: Bayer UK Ltd, Bayer House, Strawberry Hill, Newbury, Berks RG13 1JA, UK. Tel.: 0635 39518.*

### On-line effluent monitoring leaflet

Low maintenance TC and TOC effluent monitors are featured in a new leaflet published by Ionics.

The Ionics 6800 series are on-line instruments for continuous automatic monitoring of plant effluents, raw/feed waters, processing cooling waters, condensates and treatment water intakes. They are suitable both for determining the legal limits for effluents and for efficiency studies on plant processes.

Designed for continuous use and low maintenance, the Model 6800 TOC system a unique fluidic carbonate removal system with no pumps nor moving parts. Built into this is automatic sample dilution, which is ideal for high load/high salt applications. The system can sample multiple streams and features automatic calibration, alarms and control outputs.

The 6800 series instruments have a wide scale range from 0–2 ppm to 0–30 000 ppm. Cycle times are 2.5 min for TC and 5.5 min for TOC.

*For a free copy of the new leaflet contact Ionics UK Ltd., Carrington Business Park, Carrington, Urmston, Manchester M33 4DD, UK.*

### Flue gas analyser systems

A new brochure from Teledyne Analytical Instruments describes the series 9000 Flue Gas Analyzer Systems. These optimize combustion efficiency, minimize exhaust emissions and can be used in every industrial process that burns fuel. These machines can be customized to suit exact specifications.

All Series 9000 Systems utilize continuous extractive sampling methods that pull sample from the stack and provide near-line monitoring of the flue gas content. Simple, reliable air aspirators or rugged sampling pumps are part of a complete Series 9000 sampling system. Teledyne also provides accessories, including probes, preconditioners and sample lines.

*Oxygen.* All Series 9000 Analyzers feature oxygen analysis using Teledyne's patented Micro-Fuel Cell. This reliable sensor is a sealed electrochemical transducer with no electrolyte to change or electrodes to clean, so it is virtually maintenance-free. Because it has an absolute zero, no zero gases are needed for calibration. Air (20.9% $O_2$) is used for span purposes.

*Combustibles.* A low temperature catalytic bead sensor provides reliable measurement of total combustibles in the Models 9700 and 9750. Standard range of analysis is 0–5% CH4 equivalent (other ranges available).

*Carbon monoxide.* Two methods of CO analysis are available. The low cost Models 9100 and 9150 use a longlife, disposable electrochemical sensor for accuracy and reliability.

A non-dispersive infrared analyzer is an alternative approach to CO monitoring that is employed in the Model 9300. This infrared analyzer is a dual-path, single source device that uses optical techniques to eliminate potential interferences. The analyzer features a solid-state vibration-resistant detector for accurate and reliable operation and an automatic zero circuit for long-term stability.

*Carbon dioxide.* Optionally available in the Model 9300 is a $CO_2$ analysis. Standard range of this analysis is 0–20% $CO_2$ (others available). An infrared analyzer is used to measure $CO_2$, and it provides the same outstanding features and performance as the Model 9300's CO infrared analyzer.

The Model 9600 Oxygen ($O_2$) Analyser System can be used with boilers, furnaces and process heaters that are fired with such clean fuels as natural gas and low sulphur fuel oils. It uses a simple sample system centred around a highly reliable air aspirator. The system automatically and continuously conditions flue gas for accurate analysis. The Model 9600 is compact, easy to use and simple to maintain.

The $O_2$ analysis is accomplished with Teledyne's proven Micro-Fuel Cell sensor. The Model 9600 features three ranges of analysis: 0–5%, 0–10% and 0–25% $O_2$. Other features include a local meter readout, fibreglass enclosure and corrosion-resistant wetted parts. Battery- or AC-powered systems are available.

### Portable combustion efficiency analyser

Teledyne's MAX Portable Combustion Efficiency Analyser measures four flue gas parameters (oxygen, carbon monoxide, total combustibles and temperature) and then automatically calculates net combustion efficiency. A theoretical calculation of $CO_2$ content is also provided.

The MAX features three LCD displays and a membrane keyboard that allows selective display of any measurements, calculations and other information provided by the MAX. When connected to a printer, the MAX provides a permanent record of stored data, including a print-out of its operating instructions, which are stored in permanent memory (ROM). The MAX's 'temporary' memory (RAM) handles up to 20 sets of complete data as well as all calibration settings. Integral battery back-up of the RAM prevents

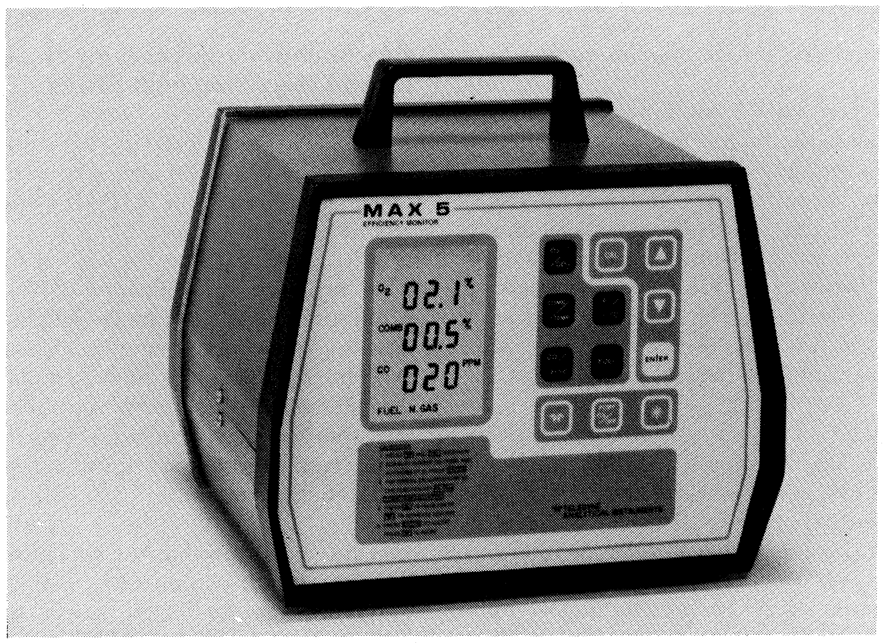

*Teledyne portable combustion efficiency analyser.*

loss of calibration data when the MAX is turned off.

The MAX also features automatic calibration of the $O_2$ analysis and auto-zero for the CO and combustibles measurements. This means that calibration is fast and no zero gases are required. Proven sensor technology assures high accuracy and reliable operation.

Powered by rechargeable Ni-Cd batteries, the MAX is a completely self-contained unit. Everything needed to measure efficiency is standard including: sample probe with Type K thermocouple; flowmeters for sample and calibration gases; sampling with mini-pump and condensate trap; battery recharge circuit; and an RS-232C output connection for the optional printer.

*For further information contact: Teledyne Analytical Instruments, The Harlequin Centre, Southall Lane, Southall UB2 5NH, UK.*

### Continuous monitoring of trace amounts of oxygen

A new brochure from Teledyne Analytical Instruments describes the series 300 range of analysers designed for continuous monitoring of trace or per cent amounts of oxygen in potentially hazardous environments. Each unit is designed to meet the European operating standards set by Cenelec (Comite European de Normalisation Electrotechnique).

The Model 316R Trace Oxygen ($O_2$) Analyser monitors $O_2$ contamination in nitrogen, argon, helium and many other pure gases and gas mixtures. The Model 316R accurately monitors oxygen from ppm to ppb levels. The Model 316R can be used in process control, quality assurance and process protection in a wide variety of industrial applications.

The heart of the Model 316R is Teledyne's highly accurate Micro-Fuel Cell $O_2$ sensor. The Model 316R features four full-scale switch-selectable ranges: 0–10, 0–100, 0–1000 and 0–10 000 ppm $O_2$. Also available are optional ranges as low as 0–1 ppm $O_2$. Other features include a special span range for air calibration, signal output, integral meter readout and optional alarms.

The Model 306WAM On-line Trace Oxygen Analyser guards against $O_2$ contamination in hydrogen, nitrogen, argon and other ultra-pure gases used in the manufacture of high density VLSI and VHSIC devices. The Model 306WAM offers full scale analysis as low as 0–500 ppb $O_2$ with an accuracy of $\pm 1\%$ and a sensitivity of 1%.

The high accuracy and sensitivity of the Model 306WAM makes it useful in a variety of applications. In the air liquefaction industry the Model 306WAM monitors $O_2$ contamination at several points in the manufacture of pure gases. In gas delivery and storage areas, the Model 306WAM can assure that the correct grade of gas is received. At the point of use, it can help find leaks.

The Model 306WAM uses Teledynes Micro-Fuel cell sensor and also features three switch-selectable ranges, meter readout, signal output and alarm capability. Standard housing for the Model 306WAM is general-purpose and panel mounted; explosion-proof housings are available.

*For further information contact: Teledyne Analytical Instruments, The Harlequin Centre, Southall Lane, Southall UB2 5NH, UK.*

### Brochure describes enzyme kinetics and reaction model-fitting

Hewlett-Packard Company has published a new, eight-page, free brochure describing the HP enzyme kinetics system.

The brochure (Publication 5956-4182) describes how more reliable results can be obtained from fewer experiments. It shows, for example, that all the data required for optimizing complete experimental procedures can be obtained from a few initial measurements.

The system featured in the brochure has three main components: a software package dedicated solely to enzyme kinetics; the latest HP diode-array UV/visible spectrophotometer; and an HP UV/vis ChemStation. The system automates the study of enzyme mechanisms from data acquisition, through evaluation and model-fitting, to the printing of results.

For determining rate data, up to six assays can be carried out in parallel. The brochure lists four curve-fit models for calculating rate data; alternatively, users can enter their own equations.

For reaction model-fitting, the brochure describes how a rate-data table can be compiled using data from previous experiments, or from other sources. Nine enzyme-reaction models are listed: they include models with single substrate with or without inhibition and with two substrates.

Again, users may enter their own equations. Other examples include showing how the fit of the experimental data with the models can be interpreted from graphical and numerical reports.

*For further information contact: Hewlett-Packard, Route du Nant-d'Avril 150, P.O. Box, CH-1217 Meynin 2, Geneva, Switzerland.*

### Support for scientific authors

Thousands of scientific references are available at the touch of a key with the new Reference Manager scientific software program. This database management program retrieves full references from 20 000 entries in less than 5 s, and allows a scientific or medical author to store up to 32 000 bibliographic references. Reference Manager software can be used with IBM PC/XT/AT and PS/2 personal computers, and microcomputers that support MS/PC-DOS operating systems.

Reference Manager has a fixed format that is of particular benefit when transferring a file between colleagues jointly writing a scientific article but located at separate facilities.

*For further information contact: Beckman Ltd, Progress Road, Sands Industrial Estate, High Wycombe, Bucks HP12 4JL, UK. Tel.: 0494 41181.*

### CCL launch computer-based translating system

Communication Control Ltd (CCL), a subsidiary of Siemens Ltd, has just launched its METAL computer-based translating system.

The system has been developed by the Siemens Application Software Division in Munich, and German has so far been the primary source language for the development of language pairs. Further language pairs providing combinations of English, German, French, Spanish and Dutch will be launched over the next 2 years.

METAL is designed to translate technically oriented text such as quotations, tender requirements, tender bid documents, technical user guides, operating manuals, service handbooks, parts lists, technical journals and technical publications. METAL therefore has a broad range of applications for industry, commerce, public services and large translation bureaux.

METAL works at a speed of one word per second, and can translate 200 pages per day. In the past, translation systems have rendered texts word for word without analysing meaning. The result was, because rules of syntax and complexities of semantics were ignored, usually nonsense. METAL analyses the entire sentence before determining the meaning of the individual sentence components.

Full integration into the office automation environment is provided by the use of the UNIX based Siemens MX2 computer. Artificial intelligence resides on a LISP shell connected via Ethernet communications.

METAL requires post-editing; its function is to assist, not replace, human translators. It relieves the drudgery of translating hundreds of pages of routine words before the skilled interpretive input of the translator is required.

*For further information contact: Communication Control Ltd, 854 Brighton Road, Surrey CR2 2UX, UK. Tel.: 01 660 1118.*

### Further sponsorship of computer-aided chemistry course at Surrey University

There is no doubt that future major advances in the application of computer technology will stem from scientists versed in computing rather than computer experts interpreting the requirements of scientists.

For the analytical chemist, there is an immediate requirement to become conversant with computer programming, interfacing and database technology, all of which will allow more extensive use to be made of data produced by analytical instrumentation.

Unfortunately, the success of this initiative is likely to be compromised by the relatively poor representation of analytical chemistry in UK tertiary education with only five centres concentrating on higher degrees. As a result, the demand of industry for trained analysts, particularly at first degree level, vastly exceeds supply.

The staff of the chemistry department at the University of Surrey recognised this problem some 3 years ago and proposed a solution in the form of a 4-year BSc course in Computer-Aided Chemistry.

The course was designed and implemented by Professor Jones and Drs Buist and Povey, with the aim of producing graduates whose training would reflect the dynamic multi-disciplinary needs of the UK chemical industry. Great care was taken to ensure that the extension of the syllabus to include computational skills would not compromise the chemistry content.

At an early stage, the organisers approached industry for both direction and sponsorship to ensure that the tuition and equipment used in practical sessions remained current.

Initial sponsorship for the course was from Perkin-Elmer who, in addition to funding an annual student scholarship, also provided a Chromatographics III data station based on the 7000 series computer together with software for handling data from gas and liquid chromatographs.

The industrial base of the course sponsorship has now been extended by the involvement of Glaxo and Hewlett-Packard.

Hewlett-Packard have provided six Vectra workstations configured in a Local Area Network (LAN), together with a Hewlett-Packard 8452 diode array spectrophotometer and Chemstation to a total value of £90 000.

The cost of installation and maintenance of the analytical hardware donated by Hewlett-Packard will be borne by Glaxo, who are among the pioneers of linear diode array spectroscopy applications. Glaxo will also provide training for both course supervisors and students to ensure that the practical sessions reflect current applications of the technology.

Alan Williams, Perkin-Elmer Product Manager for Laboratory Information Systems (LIMS) stated: 'Perkin-Elmer is pleased to be joined by Hewlett-Packard and Glaxo in the joint sponsorship of this important new course'.

In the words of Ken Leiper, Manager of Glaxo's Analytical Development Division: 'The donation and liaison programme will ensure that the course tutors are kept aware of industry's changing requirements for high quality information and, in particular, how this need may be met effectively by the novel use of computer-aided analysis systems.'

Dave Aslin, General Manager of Hewlett-Packard Analytical Group in the UK, added: 'The specific applications of linear diode spectroscopy and the enormous potential of local area networks are currently under-exploited in the UK industry. The University of Surrey's commitment to training personnel in the imaginative use of these systems in the laboratory is well deserving of our support.'

For the University of Surrey, Professor John Jones commented: 'The support now being offered will do much to enable the department to meet its aim of providing well qualified graduates who, equipped with the necessary skills to meet the needs of industry, will have excellent employment prospects and will do much to enhance the future development of analytical chemistry.'

### Philips Chair in Analytical Chemistry: New appointment at Strathclyde University

David Littlejohn is the first incumbent of the Philips Chair in Analytical Chemistry at the University of Strathclyde. The new Chair, established through an endowment of £125 000 from Cambridge-based Philips Scientific, is a major contribution to a field which faces severe skills shortages in the UK.

Aged 35, Professor Littlejohn was previously a senior lecturer in the university's Department of Pure and Applied Chemistry. He has a long association with Strathclyde, gaining a first class honours degree in chemistry and subsequently his PhD there.

After working in industry he returned to the university in 1981 to take up the Pye Foundation Lectureship, set up as the result of a grant jointly awarded to the late Professor John Ottaway and Mr Len Morris, advanced development manager at Philips Scientific (formerly Pye Unicam).

A collaborative programme was established in order to develop low cost instrumentation for rapid multi-element analysis, and to promote and teach atomic absorption spectroscopy within the academic sphere.

The most recent result of that collaboration was the development by the university of the furnace autoprobe, which forms a key part of Philips Analytical's PU9400 series of atomic absorption spectrometers. The probe allows elemental analysis at greater levels of sensitivity than previously possible; it can also cope with many complex sample matrices.

Commenting on his appointment Professor Littlejohn said: 'As well as consolidating research in many current university projects the Philips Chair will provide opportunities to extend into new areas of analytical chemistry. A major new project will involve the development of instrumentation for the separation and detection of organic compounds present in complex mixtures and biological samples.'

*For further information contact: Keith Andrews, Philips Scientific, York Street, Cambridge CB1 2PX, UK. Tel.: 0223 358866, or Margaret Robertson, University of Strathclyde. Tel.: 041 552 4400, ext. 2182.*

### Upgraded laboratory processing methods with PC-Lab

New on the market from Spectrum Computer Services plc is the updated computer-controlled and automated reactor PC-Lab, designed to make expensive and time-consuming laboratory processing tasks both more reliable and less labour-intensive.

The system facilitates the running of most common laboratory processes, including addition of reactants, temperature control for heating, cooling or isothermic conditions, reflux and distillation from vacuum to ambient pressure. Controlled addition of reactants can be determined by time, temperature or pH levels.

The system comprises a reactor assembly unit, an Olivetti PC, control valves and balance, plus temperature and pH probes. Control equipment and monitoring probes are linked to the PC and all reaction results are fed into and stored by the system, enabling tests to be re-run, evaluated or adapted at the touch of a button.

Improvements to software cover the inclusion of comprehensive reporting facilities, with extensive manipulation options allowing data gathered in the system to be easily transported to the user's own preferred spreadsheet format.

In addition, PC-Lab offers a built-in series of safety features giving a continuous self-monitoring facility. The unit runs automatic checks at the start of each test to ensure that all relevant pieces of equipment are attached and functioning. Procedures for shutdown can be defined by the operator to suit the test, and cover, for example, drainage of flask or 'dumping' of items into the flask to kill reactions.

Spectrum provide full customer support and technical back-up, including specialised hardware and software options. A comprehensive initial training programme has been developed for prospective operators, and the company offers a full installation and servicing contract as part of the package.

*For further information, contact: Spectrum Computer Services plc, Spectrum House, East Parade, Bradford BD1 5RJ, West Yorkshire, UK. Tel.: 0274 308188.*

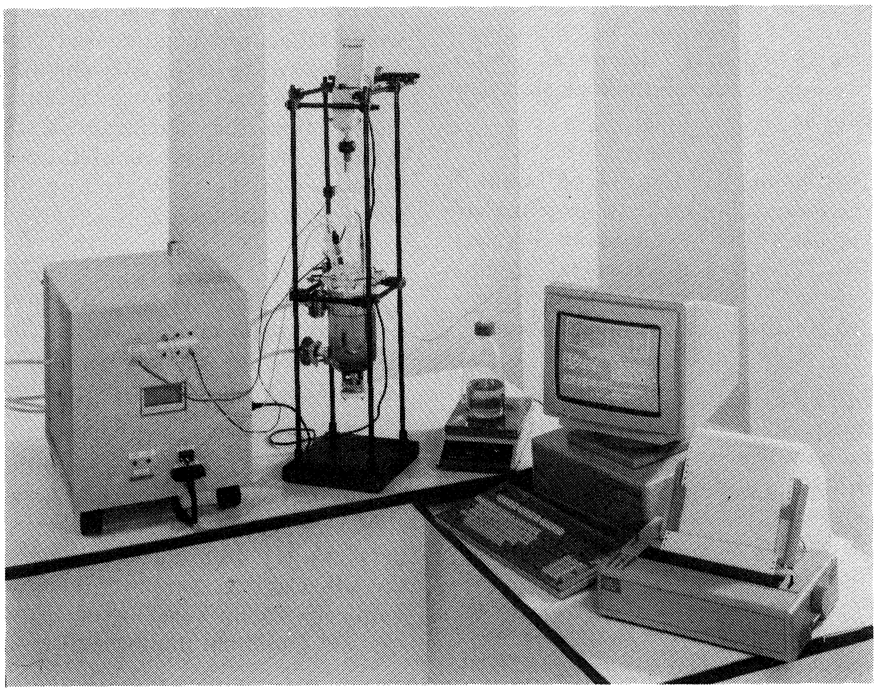

*PC-Lab, the computer-controlled reactor from Spectrum Computers.*

### Lotus Measure provides the direct link between data acquisition and reporting

Lotus Measure software has been introduced by Industrial Data Processing to speed up and make accurate the collection of analysis data from instruments and sensors. The package, for IBM and compatible PCs, provides a direct and low cost link from instruments operating via the IEEE-488 bus or an RS-232C serial interface, and plug-in Data Acquisition boards, into a Lotus 1-2-3 spreadsheet.

In all three interface environments, Lotus Measure saves the user from time consuming custom programming, and eliminates the need for data to be entered by hand, or imported from other programs for analysis and reporting. Settings sheets within Measure allow the user to set up interface configurations and programming commands for instruments, or settings such as input data sources and sampling rates from A/D boards, while providing the means of storing a particular set up for any experiment so it is immediately and identically repeatable.

There is also an extended range of macro commands, as the original 1-2-3 advanced macro command language is supplemented by new commands specific to Measurement tasks. By combining Measure menu choices with macro commands, the user may automate a custom application in any of the IEEE-488, RS-232C or Interface Board environments.

In this way, Lotus Measure allows experiments to be controlled under strict scientific method. Data may be collected, analysed and stored under one set of conditions, and then the experiment may be repeated with just one parameter changed. Uncertainty over which conditions have altered is removed. This same feature allows, for example, a sequence of different tests to be easily called up and processed on an instrument bank in an Automatic Test station.

When used with plug-in data acquisition boards, Lotus Measure lets the user program linearisation and other conversion formula into 1-2-3 spreadsheet cells so that raw A/D units can be translated straight into engineering values of temperature, strain, and so on.

Up to 64 channels of data may be acquired concurrently, and there is a facility to display up to 16 channels of incoming data in real time. All inputs are collected and stored directly into a 1-2-3 worksheet.

Lotus Measure costs £395 for all IBM PC compatibles

*For further information, contact: Industrial Data Processing Ltd, Unit G2, Melbourn Science Park, Melbourn, Nr Royston, Herts SG8 6TB, UK. Tel.: 0763 62662.*

### New electronics add transmitting and alarm features to flowmeter range

New electronics which facilitate transmitting and alarm features on its established range of CMI flowmeters has been announced by Platon Instrumentation.

The all-metal, direct-reading flowmeters with magnetically coupled indicators measure the flow of liquids and gases at pressures up to 350 bar and temperatures up to 200 °C. The flowmeters can incorporate an electronic transmitter or high (rising flow) and/or low (falling flow) alarm, facilitating measurement and/or alarm signals in a control room which is remote from the main system.

Type CMI is the base model. CMI/E is a micro-processor based, two-wire electronic flow transmitter. This instrument requires a 12 or 24 V DC supply and will provide a 4–20 mA output into a 600 Ω (max) load, which is directly proportional to the flow being measured. A differential capacitor serves as the transducer which is magnetically coupled to the float giving the instrument high temperature stability. Housed in a polyester-coated environmentally sealed box are all the associated electronics, including the micro-processor, power supply, digital/analogue converter, transducer assembly, oscillators and output circuitry.

Type CMI/A offers alternative single or dual channel alarm functions. Inductive proximity sensors are used for intrinsically safe circuitry as required in chemical or petrochemical applications. An infra-red retro-reflective two-wire device is offered as standard, with alarm condition at 4 mA relay de-energised, or 20 mA relay energised. The electronics and the enclosures have been designed for reliability and to withstand the most arduous service conditions and environmental situations. Housings have been tested and certified to IP65 standard.

*For further information, contact: Platon Instrumentation, Platon Park, Viables, Basingstoke, Hampshire RG22 4PS, UK. Tel.: 0256 470456.*

### Mettler SQC61 Quality Control System

The Mettler SQC61 Quality Control System helps to save costs by reducing overfilling to a minimum and by detecting and reporting underfilling immediately; homogeneous filling assures a constant standard of quality of the products.

The SQC61 system enables comprehensive documentation of the product to be printed out by the operator. The system takes national tolerance systems, including pharmacopeia, into account.

The SQC61 system can be used as a manual, semi-automatic or fully automatic weighing station. Both single and multiple weighing stations are available. Data for all recorded values are saved on cassette or disk.

The user has available a wide selection of parameters for the allocation of sample data. This includes sample designation and number, minimum fill quantity, density (for samples defined by volume), different tolerance systems, different tare inputs random sample size, various parameters for adjustment messages, weighing modes, etc.

The SQC61 system also offers a high degree of flexibility with regard to the evaluation. The use of two printers of different character width (80/132)

simplifies the documentation as separation of statistical and sample data improves the overview. A mean value trace gives information on the behaviour of the filling process for up to four different samples at the same time. A histogram illustrates the statistical production data; this can be displayed at any time over various, freely definable periods.

*For further information, contact: Mettler Instrumente AG, CH-8606 Greifensee, Switzerland.*

### Automation for microplate assays

The new Biomek SL is a labware loading option for the Biomek 1000 automated laboratory workstation. The SL is capable of transferring labware and pipette tips to and from the Biomek 1000 tablet without operator intervention. It consists of a robotic arm, stack area with up to six stacks and mounting hardware to position the system in conjunction with the Biomek 1000. Up to three stack areas (a total of 18 stacks) provide maximum system capacity.

Individual stacks in each stack area hold labware to be loaded and three sizes are available: an eight-shelf version for multiwell plates; a four-shelf stack for P-250 tips, the Biomek reservoir system and minitubes; and a three-shelf unit for P-1000 tips and test-tubes.

Each stack is made to be interchangeable with any other of the same configuration and stack shelves are clearly identified by a numerical marking on the outer surface. Positive locating plates align stacks in the exact position for easy, error-free placement and removal.

The software package used for the Biomek 100 also operates the Biomek SL and, once the content of each stack has been defined, minimum commands are necessary to perform virtually any loading operation. A built-in checking facility eliminates the possibility of incompatible labware configurations being used.

No complicated teaching routines are needed for the Biomek software due to positive positioning of the various stacks, and all positioning and location commands are known to the system.

The Biomek SL utilises a tactile sensor in it's hand mechanism which allows it to 'feel' what is being gripped. All loading sequences are verified by this sensor which facilitates the removal and replacement of lids on multi-well plates. An alarm is also included to alert the operator in the event of a load sequence failure or the absence of labware at a particular location.

*For further information, contact: Beckman Ltd, Progress Road, Sands Industrial Estate, High Wycombe, Bucks HP12 4JL, UK. Tel.: 0494 441181.*

### Skalar New Model Autosampler

Skalar has released the New Model SA 1070 Autosampler, developed for the Skalar range of automatic analysers. The SA 1070 features: a capacity of 250 samples and eight standards; a sample volume variable from 1 to 15 ml; two different samples picked up simultaneously; local control or control via external computer; random access sampling, *X–Y* mechanism; automatic dilution and re-run of over- range of samples and samples with high carry over; and automatic start-up and shut-down.

The autosampler is suitable for high throughput of variable samples. Laboratory operating hours can be extended by overnight runs with any over-range samples automatically diluted and re-run.

*For further information, contact: Skalar Analytical BV, Spinveld 62, 4815 HT Breda, The Netherlands.*

